# Parallel Implementation of Nonadditive Gaussian Process Potentials for Monte Carlo Simulations

**DOI:** 10.1021/acs.jctc.3c00113

**Published:** 2023-06-27

**Authors:** Jack Broad, Richard J. Wheatley, Richard S. Graham

**Affiliations:** † Molecular Foundry, Lawrence Berkeley National Laboratory, Berkeley, California 94720, United States; ‡ School of Chemistry, University of Nottingham, Nottingham, NG7 2RD, England; ¶ School of Mathematical Sciences, University of Nottingham, Nottingham, NG7 2RD, England

## Abstract

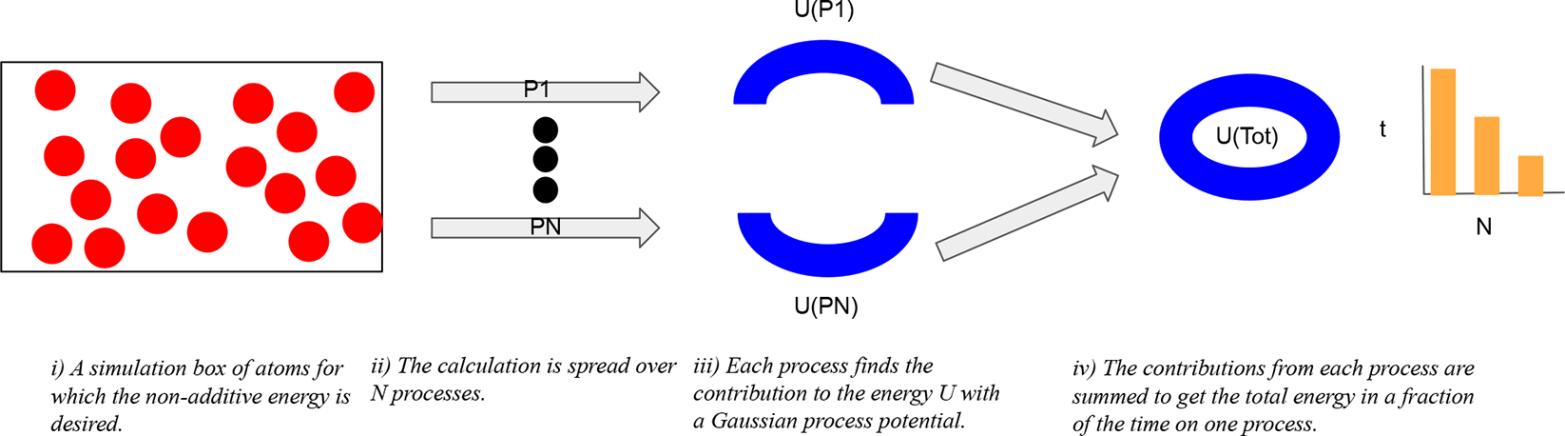

A strategy is presented to implement Gaussian process potentials in molecular simulations through parallel programming. Attention is focused on the three-body nonadditive energy, though all algorithms extend straightforwardly to the additive energy. The method to distribute pairs and triplets between processes is general to all potentials. Results are presented for a simulation box of argon, including full box and atom displacement calculations, which are relevant to Monte Carlo simulation. Data on speed-up are presented for up to 120 processes across four nodes. A 4-fold speed-up is observed over five processes, extending to 20-fold over 40 processes and 30-fold over 120 processes.

## Introduction

First-principles predictions of macroscopic properties of fluids have the potential to improve models in a wide range of problems, from climate change^1,2^ to water desalination.^3^ Such predictions require a potential energy surface (PES), which is usually obtained by interpolating *ab initio* calculations of the molecular interactions and a method to convert this PES into macroscopic predictions. Though virial equations of state^4^ can predict properties of gases, molecular simulation^5,6^ is required for liquid and solid properties. For a simulation to achieve quantitative accuracy, it requires a potential that makes predictions of *ab initio* quality of the underlying microscopic interactions.

There are many strategies to fit potentials to *ab initio* data including parametric fits in which the parameters of the potential are postulated *a priori*. Recent examples include *ab initio* potentials,^7−12^ which are so-called as they employ functional forms motivated by first principles. However, a parametric model cannot capture completely the high-dimensional PES. Furthermore, modeling mixtures with *ab initio* potentials is cumbersome, requiring fitting of many potentials with varying numbers of parameters to different data sets. These potentials also often fail to capture accurately three-body nonadditive effects, which are significant in predicting properties of liquids and solids.

Machine-learned potentials (MLPs)^13,14^ offer a route to first-principles predictions in simulations through nonparametric interpolation of quantum-mechanical data. Nonparametric interpolation permits high-accuracy approximations of even a three-body PES without experimental data. MLPs therefore have the capacity to facilitate quantitatively accurate simulations, which could reduce the need for experiments when calculating gas, liquid, and coexistence properties.

MLPs employ different methods of prediction, with Gaussian processes^15^ (GPs) and neural networks (NNs) being common choices. Recent developments in the training of NN potentials have reduced the number of training points they require to be comparable with that of GP potentials.^16,17^ However, to avoid overfitting of a NN potential when the distribution or number of training points varies (such as when fitting to a different PES) may require alterations to the number of hidden layers or neurons per layer.^18^ Overfitting is not a concern when training GPs, meaning the exact same algorithm used to train a GP potential on one system can be applied to other systems with no alterations.^19^ Consequently, GP potentials are ideal for the simulation of mixtures.

GP potentials have been produced for various systems^20−38^ and achieve accuracies equivalent to NN potentials.^18,24,39^ GP potentials have also been trained via transfer learning,^40,41^ in which a training set is selected from a larger reference set of relatively cheap *ab initio* calculations and upgraded to a higher level of theory. Thus, a GP potential from coupled cluster calculations can be produced with a few expensive calculations.

GP potentials are unique in offering high accuracy interpolation with small training sets and flexible training algorithms. However, prediction with GPs is computationally intensive, scaling linearly with the training set size. As this can reach ∼1000 points for nonadditive PES of even small systems, effective parallelization algorithms for GP potentials are important. A general parallelization algorithm is presented here, with effective parallel speed-up demonstrated for nonadditive energy calculations of the type required in Monte Carlo (MC) simulations. This algorithm extends straightforwardly to additive energy calculations and comprises two parts: the precalculation of the covariance function using shared memory and the distribution of triplets over processes. Though the precalculation is GP-specific, the distribution of the triplets is not restricted to any potential. Moreover, it is effective in ensuring that the triplets are disseminated equitably across processes.

Efficient GP potentials would facilitate quantitatively accurate molecular simulations, which have wide-ranging applications. For example, the US government anticipates molecular simulations will be instrumental in carbon capture, utilization, and storage (CCUS).^42^ CCUS pipelines contain small molecules such as N_2_, O_2_, Ar, and H_2_ in addition to CO_2_,^21^ and GP potentials have modeled interactions between various small molecules reliably.^20,22,23^ Thus, GP potentials are ideal for simulations to determine, with quantitative accuracy, properties of the mixtures in CCUS pipelines. This would be achieved without experiment and would enable liquid-phase predictions, which are impossible by current approaches.

The following sections discuss a method for efficient implementation of GP potentials, starting with the background theory in section 2. This is followed by an overview of the full box and atom displacement calculations in section 3. Thereafter a summary of the computational details is given in section 4, before the results are presented in section 5.

## 2 Background Theory

### Gaussian Process Potentials

All GPs employ a kernel function when making predictions, which maps the problem being modeled to a feature space defined by the covariance between points. The GP potentials herein use a symmetric squared exponential kernel *k*_Sym_,^20,22,23^
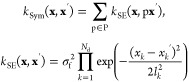
1Here, *k*_SE_ is the squared exponential kernel, **x** and **x***′* are vectors of inputs, *N*_d_ is the number of dimensions in these vectors, and P is the group of permutations of **x***′* under which the output is invariant. The hyperparameters of the kernel shown in eq 1 are the signal variance σ_f_^2^ and the length scales *l*_*k*_.

Hyperparameter values are obtained by maximizing the log-likelihood log(L) of the model on the training set, where
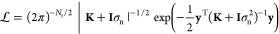
2Here, *N*_t_ is the number of training points, **I** is the identity matrix, **K** is the covariance matrix obtained by evaluating *k*_Sym_ and **y** is a vector of observations. In this work, the observations are nonadditive energies for Ar_3_ triplets, obtained at the CCSD(T) level with an aug-cc-pVTZ basis set. σ_n_^2^ is the Gaussian noise variance, which is an additional hyperparameter that is optimized. The process of developing the training set and obtaining the corresponding hyperparameters is referred to as training. Following training, predictions are made using the Woodbury vector **λ**,
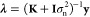
3(The Woodbury vector is denoted here as **λ** despite being typically represented as **α**, because α later denotes an atom in a triplet.)

When modeling PES, inverse interatomic distances are effective inputs for the kernel function.^20^ The symmetry-equivalence of different intermolecular configurations is incorporated into the model with the symmetric kernel *k*_Sym_, allowing training sets to be developed for the symmetry-distinct region of the phase space only. As in previous work,^20,22,23^ all data sets used to train the GP here were built with Latin hypercube sampling.^43−45^

Using a GP that employs *k*_Sym_, the nonadditive potential *U*_NA_(**x**) of a molecular triplet is
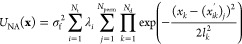
4Here, *i* sums over training points, *j* sums over permutations, *k* runs over dimensions of **x**, and (*x′*_*ik*_)_*j*_ is the *k*th coordinate in the *i*th training point, after the latter has been subjected to the *j*th permutation in the group P.

A permutation is an interchange of two or more distances, under which the energy is invariant. For example, in a triplet of identical atoms the total number of permutations in P, *N*_perm_, is six. These permutations are stored in a permutation matrix **P**, which for the three-atom example is
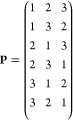
5Each element in **P** corresponds to the index of an inverse interatomic distance. Though this matrix could become large for more complex systems, its inclusion reduces the number of expensive *ab initio* calculations. If a system was too complicated to include symmetry in this way, it is possible to ignore certain permutations to reduce the size of **P**, though the effect of this is not explored herein.

## 3 Parallel Calculation of Potential Energy in Simulation

Here, a method is derived to calculate in parallel the total energy of a simulation box and the change therein following movement of an atom using a GP potential. The method exploits the functional form that describes the energy for such a potential, as shown in eq 4. Efficient parallel calculation of the total potential is achieved by distributing the molecular pairs across a number of processes and calculating the exponentials in eq 4 with shared memory. Though the exponential calculations are GP-specific, the strategy to distribute pairs is general to other potentials.

### Simulation Box Energy

The total nonadditive energy of an entire simulation box of *N*_a_ molecules, *U*_NA_^tot^, is obtained by summing eq 4 over all triplets. The full range of triplets is described generally with the molecular numbers *αβγ*, where 1 ≤ α < β < γ ≤ *N*_a_. Hence the sum over molecular triplets is
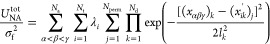
6where (*x*_*αβγ*_)_*k*_ is the *k*th inverse intermolecular distance of the triplet *αβγ*. Equation 6 therefore gives the total nonadditive energy when all triplets are calculated directly, *i*.*e*., when no cutoff distance is used.

Equation 6 does not assume any specific type for the constituent molecules, but for simplicity, it is assumed henceforth that all particles are identical atoms. Hence, *N*_d_ = 3 and the length scale *l*_*k*_ is the same for all *k*. The smallest number of exponentials required to evaluate eq 6 is identified, and their values are stored in an array. The array
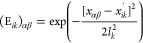
7where *i* = 1...*N*_t_, *k* = 1...*N*_d_ and α,β runs over all possible pairs, is sufficient. The loop over all training points *i* for all dimensions *k* is necessary because, for three identical atoms, all permutations of the dimensions are permitted (eq 5).

The use of a permutation matrix **P** allows one to write (*x′*_*ik*_)_*j*_ = *x′*_*iP*_*kj*__ by taking the *k*th element of the *j*th row in **P** as the index of the relevant distance in the *i*th training point. Hence
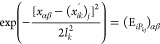
8

A mapping to identify which interatomic distance corresponds to (*x*_*αβγ*_)_*k*_, that is, the *k*th distance in the triplet *αβγ*, is required. For a triplet of atoms, **x**_*αβγ*_ = (*x*_*αβ*_, *x*_*αγ*_, *x*_*βγ*_). As *N*_d_ is small it is possible to write out the product in eq 6 explicitly and substitute in eq 8 to obtain
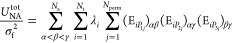
9

The algorithm in this work implements GP potentials efficiently by precomputing the exponentials in the exponential array and parallelizing the nested sum in eq 9 by distributing the triplets across processes. The algorithm to distribute pairs of atoms across processes for the sum over triplets is applicable to other three-body potentials, not just GP PES.

## Nonadditive Energy Calculation for a Simulation Box

Calculation of the total nonadditive energy of a simulation box *U*_NA_^tot^ is necessary at the outset of a Monte Carlo (MC) simulation and during any move that affects all particles, such as a volume change. To calculate *U*_NA_^tot^ necessitates evaluating  separate triplets. Though the method extends to mixtures of molecules, the subsequent discussion centers on atomic triplets of the same species such as Ar_3_, for which results are presented later. Direct evaluation of eq 4 to calculate the nonadditive energy of an atomic triplet would involve *N*_t_*N*_perm_*N*_d_*N*_a_^3^/6 exponentials, an expensive potential that scales unfavorably with *N*_a_. However, each inverse distance is common to many triplets so it is useful to precalculate exponentials once. Afterward the exponentials are assembled to calculate *U*_NA_ for any triplet.

Under periodic boundary conditions (PBCs) and a minimum image convention (MIC), *x*_*αβ*_ is the inverse minimum image (MI) distance between atoms α and β. For a simulation box of side length *L*, a cutoff *r*_c_ = *L*/2 is applied, such that if any distance in a triplet exceeds *r*_c_ then the nonadditive energy of the triplet is set to zero. The inverse MI distances are stored in array **X**.

Precalculation of the exponentials is undertaken in parallel over a total of *N*_p_ processes, each with its own rank *R*, *R* ∈ [1, *N*_p_]. The process with *R* = 1 is known as the root process. All processes may be on a single node or may be split across several nodes. Using the exponential array in parallel requires sharing large amounts of information, as all processes will likely need access to all exponentials. As the connections between nodes are not fast, the exponential array is calculated using shared memory, and each node has its own copy. Letting **E**_*αβ*_ be the segment of the exponential array pertaining to the *αβ* distance, the calculation of the exponentials on a single process proceeds by Algorithm 1. During this calculation, exponentials are calculated between pairs of atoms separated by less than *r*_c_ only.
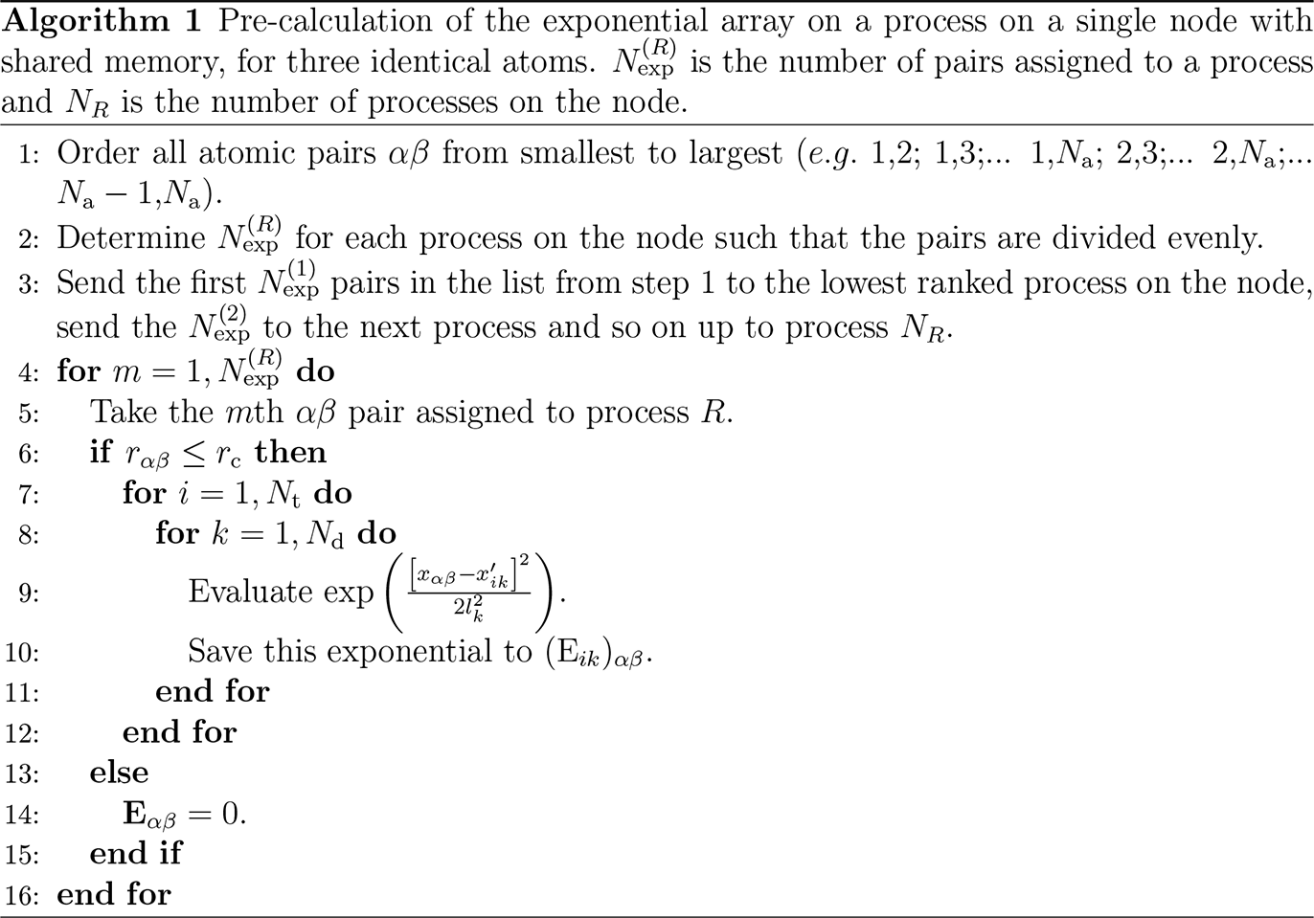


Consequently, a speed-up of a factor of up to *N*_p_ is possible if all processes are on a single node. For processes split over several nodes, the speed-up is limited to a factor of *N*_*R*_ on the node with the fewest processes.

The calculation of *U*_NA_^tot^ is split over *N*_p_ processes, reducing the number of triplet evaluations on each process by up to a factor of *N*_p_. As each process needs to only send back the total nonadditive energy of all assigned triplets (*i*.*e*., a single number) the information sharing between processes is not a limiting factor. Consequently shared memory was not required, so there is no need to consider whether processes are on the same node when evaluating the triplet nonadditive energies. The work in this section is general and is not restricted to GPs.

To allocate triplets to processes, all atom pairs in the simulation box are first divided between the processes. The pairs assigned to each process are consistent throughout the calculation. The pairs cannot be assigned in the same manner as steps 1–3 of Algorithm 1 because all pairs containing a given atom must be distributed evenly; otherwise, moving that atom would leave a process to undertake an excessive share of the calculation. An even distribution is achieved by dividing the pairs via Algorithm 2.
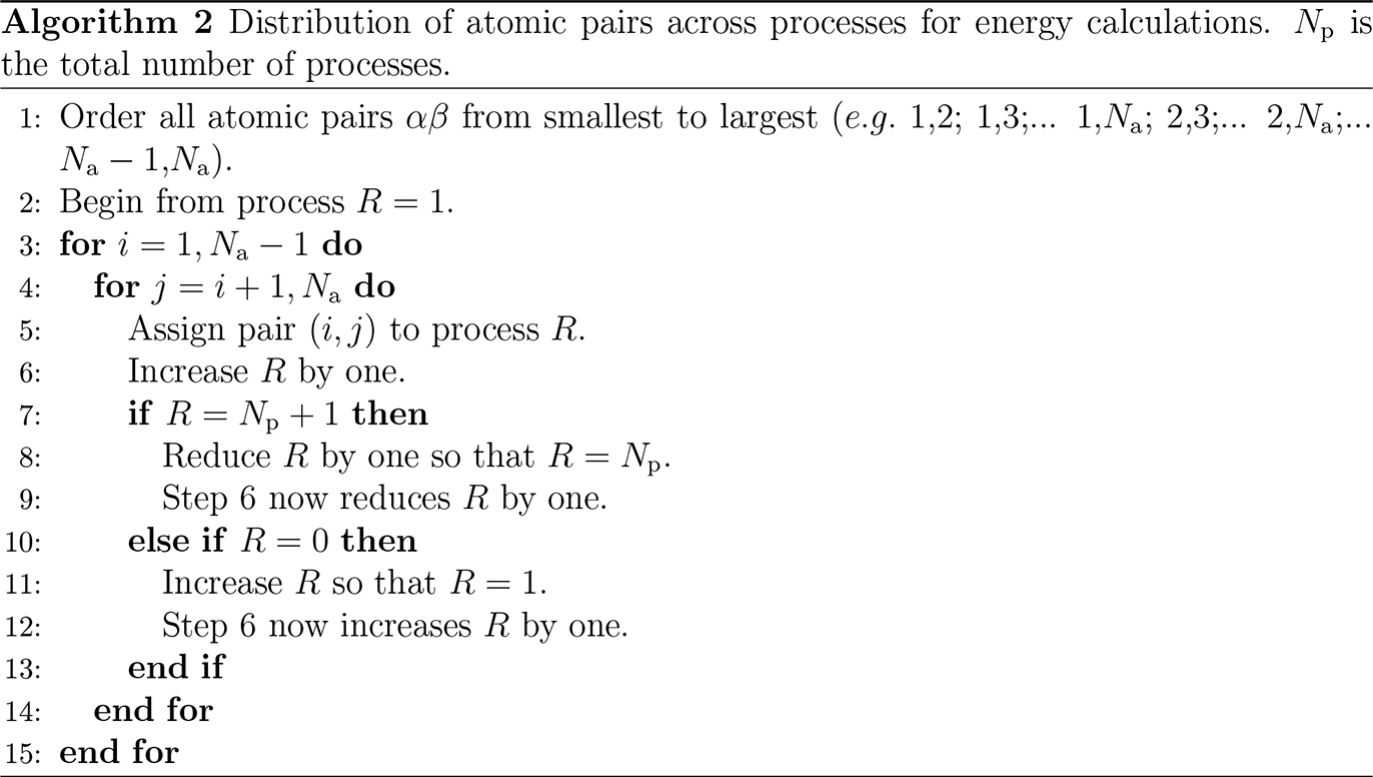


This approach for distributing the pairs over processes is general to all potentials. Furthermore, it is well-suited to the evaluation of the pair energy, as each process must calculate the energies of its assigned pairs only. Pairs are assigned in ascending order and then descending order so that when a single atom is moved, the triplets that must be re-evaluated are more equitably distributed. For every triplet, Algorithm 3 is used to verify that all MI distances in the triplet do not exceed the cutoff *r*_c_ before any calculation is undertaken.
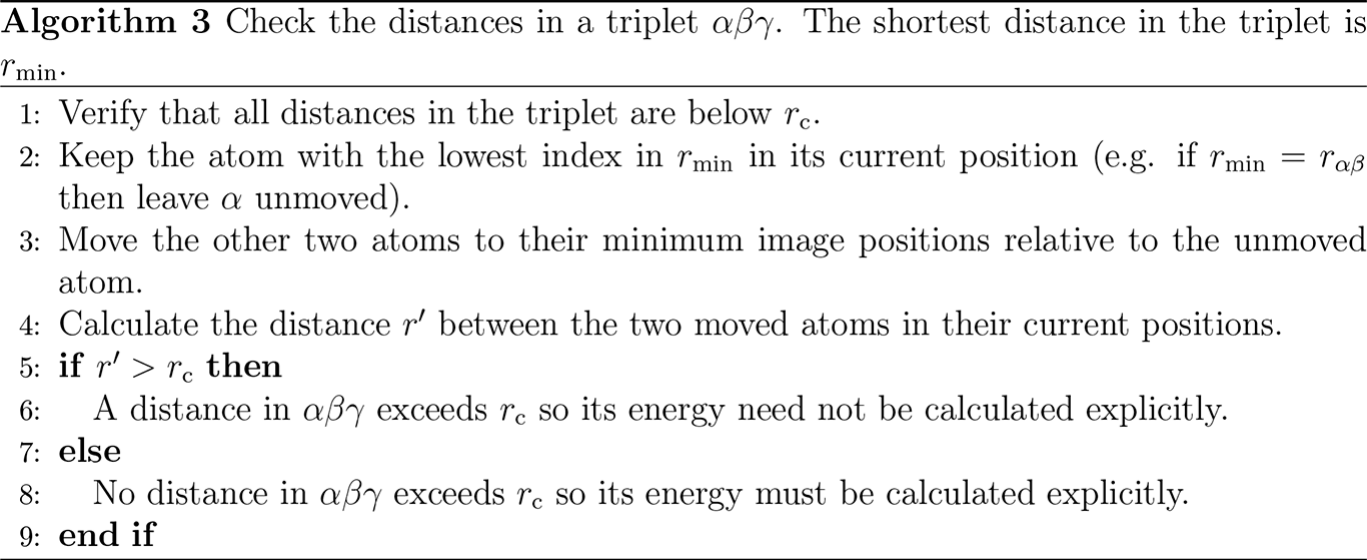


Following assignment of the atom pairs, each process calculates the nonadditive energies of all triplets α < β < γ for which it owns the *αβ* pairs. The individual nonadditive triplet energies on process *R* are stored in a vector **u**^(*R*)^, which has length equal to the number of triplets, *N*_tri_^(*R*)^, assigned to the process. The steps undertaken for the parallel calculation of *U*_NA_^tot^ are given in Algorithm 4, in which *U*_NA_^(*R*)^ is the contribution to *U*_NA_^tot^ from process *R*. Because the pairs assigned to a process are the same throughout the simulation, so are the triplets that it will evaluate.
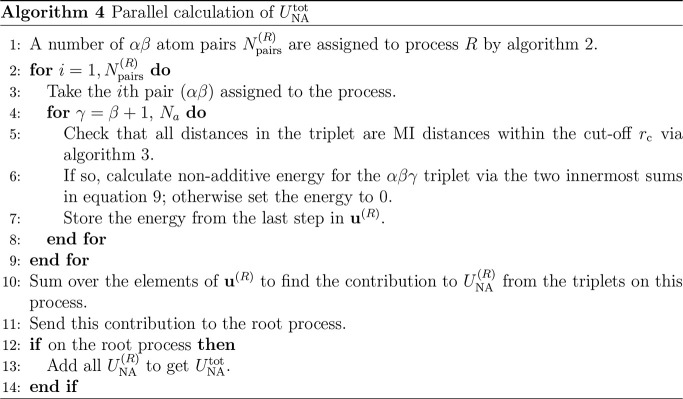


### Energy Change after Atom Displacement

Finding the new nonadditive energy of all triplets affected by moving an atom δ requires recalculation of the triplets that contain δ only, which renders it faster than the full box calculation. However, recalculation of the energy after an atom moves is undertaken more frequently during simulation. The nonadditive energy of triplets containing δ, *U*_NA_^(δ)^, are found by modifying eq 9 to give
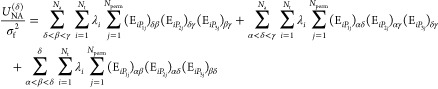
10The change in nonadditive energy Δ*U*_NA_ is found by taking the difference between *U*_NA_^(δ)^ before and after the move.

Displacement of δ alters the exponentials for all pairs in which it appears. Hence, calculating Δ*U*_NA_ for a displacement requires recalculation of the exponentials corresponding to *N*_a_ – 1 affected distances, where *N*_a_ is the number of atoms. Similarly, a Monte Carlo addition introduces *N*_a_ new pairs, necessitating a set of *N*_a_ new exponential calculations.

As Monte Carlo moves may be rejected, all old exponentials involving δ are stored. This is done separately on each process, as each process needs only save its own share of the exponentials before updating the exponential array. The exponentials on a single process are updated by Algorithm 5, where **O**_*m*_ contains all exponentials pertaining to the *m*th pair on process *R*.
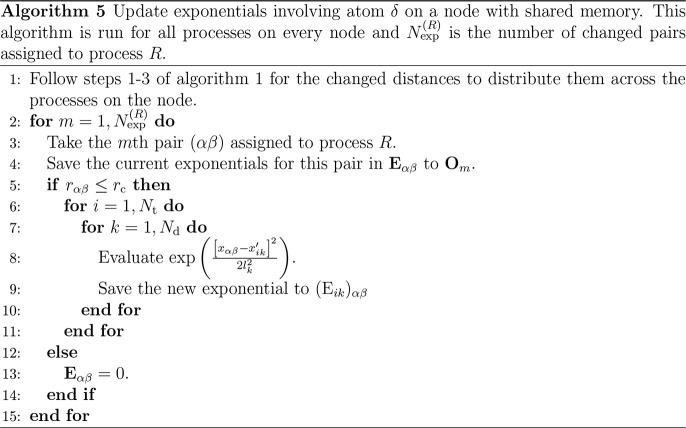


For process *R*, the change in nonadditive energy following an atom move is denoted Δ*U*_NA_^(*R*)^. Δ*U*_NA_ is therefore the sum of Δ*U*_NA_^(*R*)^ over all processes. For each process, Δ*U*_NA_^(*R*)^ is calculated from the difference between the new and old nonadditive energies for each triplet containing the moved atom δ. The new nonadditive energies of each affected triplet on process *R* are stored in the vector **c**^(*R*)^, ready to be placed into **u**^(*R*)^ if the move is accepted. The length of **c**^(*R*)^ is equal to the number of affected triplets on the process, *N*_changed_^(*R*)^. The steps taken on each process to identify affected triplets and recalculate their energies are shown in Algorithm 6.
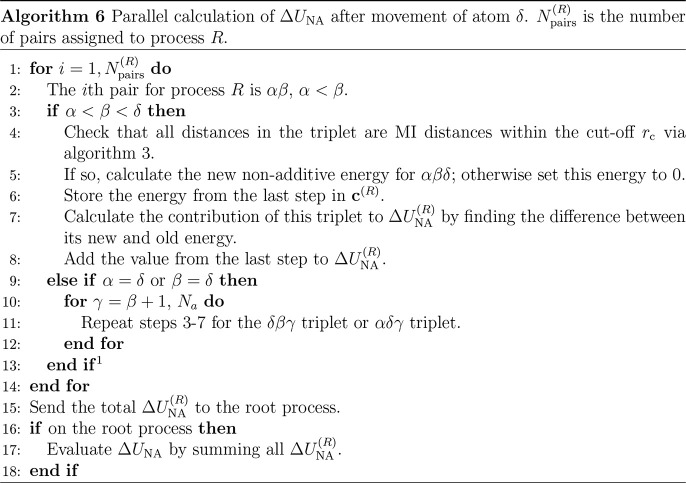
^1^If α < δ < β or δ < α < β do nothing.

The combination of all algorithms discussed thus far gives rise to a general method for parallelizing the calculation of the nonadditive energy in a simulation with a GP potential. This method is in large part general to other methods of prediction too. After a brief discussion of what must be done on accepting or rejecting a move, the full algorithm is presented in this section.

When a move is accepted in a Monte Carlo simulation, the methods outlined above require the transfer of the triplet nonadditive energies from **c**^(*R*)^ into **u**^(*R*)^. *U*_NA_^tot^, the position of the moved atom, and the inverse distances must also be updated. Meanwhile, rejecting a move necessitates replacing the exponentials in **E** with the previous ones saved in **O** from each process.

The methods presented in the preceding discussion are summarized below in Algorithm 7, which describes how the nonadditive energies are calculated for an entire simulation over *N*_moves_ moves. This algorithm includes all considerations of the periodic boundary conditions and minimum image convention, and can be generalized to exchange and volume change moves. Furthermore, other than steps 3 and 8 it is general to all nonadditive potentials.
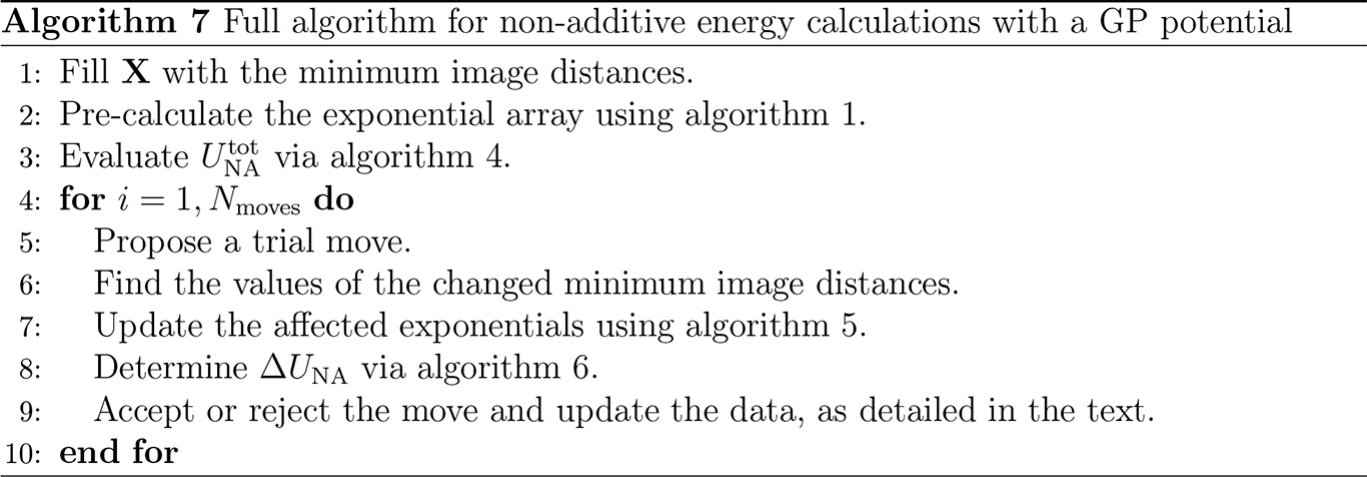


## 4 Computational Details

All calculations were undertaken for a simulation box of argon atoms to assess the speed-up possible with the proposed parallelization. Much recent work implementing *ab initio* potentials in simulation^7−12^ has focused on noble gases as exemplars due to the relative simplicity of their interactions. Argon was chosen here specifically due to the ability of GPs to model Ar_3_ triplets^22^ and their success in developing virial coefficients for CO_2_–Ar mixtures.^19^

Augusta, a University of Nottingham high-performance computer, was used for all calculations. Each node on Augusta has 2 × 20 core processors (Intel Xeon Gold 6138 20C 2.0 GHz CPU). Code was written in Fortran, using the Fortran message passing interface for parallelization and openMP for shared memory. For all calculations, the O3 optimizer flag was enabled to minimize computational time prior to parallelization.

The three body nonadditive energy was calculated for a simulation box of *N*_a_ = 500 argon atoms, which had random starting positions. The side length *L* of the box was 29 Å, corresponding to a density of 1.4 kg dm^–3^, which exceeded the critical density.

Periodic boundary conditions (PBCs) and a minimum image convention (MIC) were included, with a cutoff of *r*_c_ = *L*/2 = 14.5 Å applied to all calculations. The cutoff resulted in roughly 12% of nonadditive energy calculations being undertaken explicitly. 150 displacements were attempted, with atoms selected at random and moved up to 1.5 Å forward or backward along each of the *x*, *y*, and *z* axes. These moves were alternately accepted and rejected without the use of the Metropolis method.

The nonadditive GP potential was trained on a 999-point training set, via the method of Wheatley and Graham,^19^ in which all energies were calculated at the CCSD(T) level of theory using an aug-cc-pVTZ basis set. This potential achieved a root-mean-square (RMS) error of <1% of the RMS value of the reference set and was used to make nonadditive energy predictions of first-principles quality for all explicit calculations. Training on this set took around 7 h. The nonadditive energy of a triplet *αβγ* was calculated as
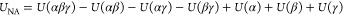
11where *U*(*αβγ*) is the total triplet energy, the negative terms are the pairwise interaction energies, and the final three terms are the energies of the individual atoms.

## 5 Results and Discussion

The factor by which the speed of a calculation increases when spread over a number of processes *N*_p_ is referred to here as the speed-up. This is defined as *t*_1_/*t*_*N*_p__, where *t*_*i*_ is the wall clock time in seconds for *i* processes. Speed-up data are presented for atom move and full box calculations.

**Figure 1:**
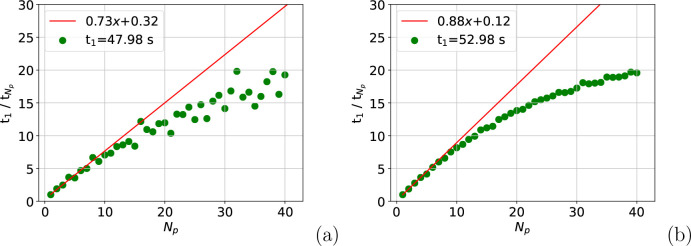
Speed-up on one node for the atom move (a) and full box (b) calculations. The move data are summed over 150 moves, of which half were accepted.

The speed-up when completing the atom move and full box calculations in their entirety, *t*_total_, is shown in Figure 1. This evidence a 7.5-fold speed-up on 10 processes for both calculations under realistic simulation conditions. On 40 processes, the speed-up is roughly 20-fold.

The speed-up on 40 processes means that 2.6 s is required to calculate the full simulation box energy and 2.4 s to displace 150 atoms. For a simulation comprising 100,000 cycles, each of which consists of one volume change and displacement of every atom, this corresponds to 12 days for a first-principles-quality simulation. This number is found by calculating the time for 100,000 volume changes on 40 processes (*i*.*e*. 2.6 s × 100, 000 ≈ 3 days) and adding it to the time taken to displace all 500 atoms 100,000 times. The time to displace one atom is 2.4/150 = 0.016 s, which means the total displacement time is 0.016 s × 500 × 100, 000 ≈ 9 days. The equivalent time to utilize a GP potential on one process is 245 days. Calculating the additive energy requires a small fraction of the time for the nonadditive energy. Due to differences in the PES being considered and the number of calculations, direct comparison of these timings to those of other machine-learned potentials cannot be made without reservation. However, the times achieved here are comparable to those elsewhere,^46^ and faster reported GP potentials^39^ featured lower cutoff values and do not consider nonadditive intermolecular interactions.

An equivalent calculation using CCSD(T) with a complete basis set extrapolation would require around one h per triplet. When *N*_a_ = 500 there are ∼20.7 million triplets, of which 12% must be evaluated due to the cutoff. This necessitates ∼2.48 million calculations, which at one h each would take over 100,000 days. Similarly, the 500 atom displacements would require over 300,000 days. Thus, the time for a single cycle with direct CCSD(T) calculations would take in excess of 400,000 days or over 1000 years.

**Figure 2:**
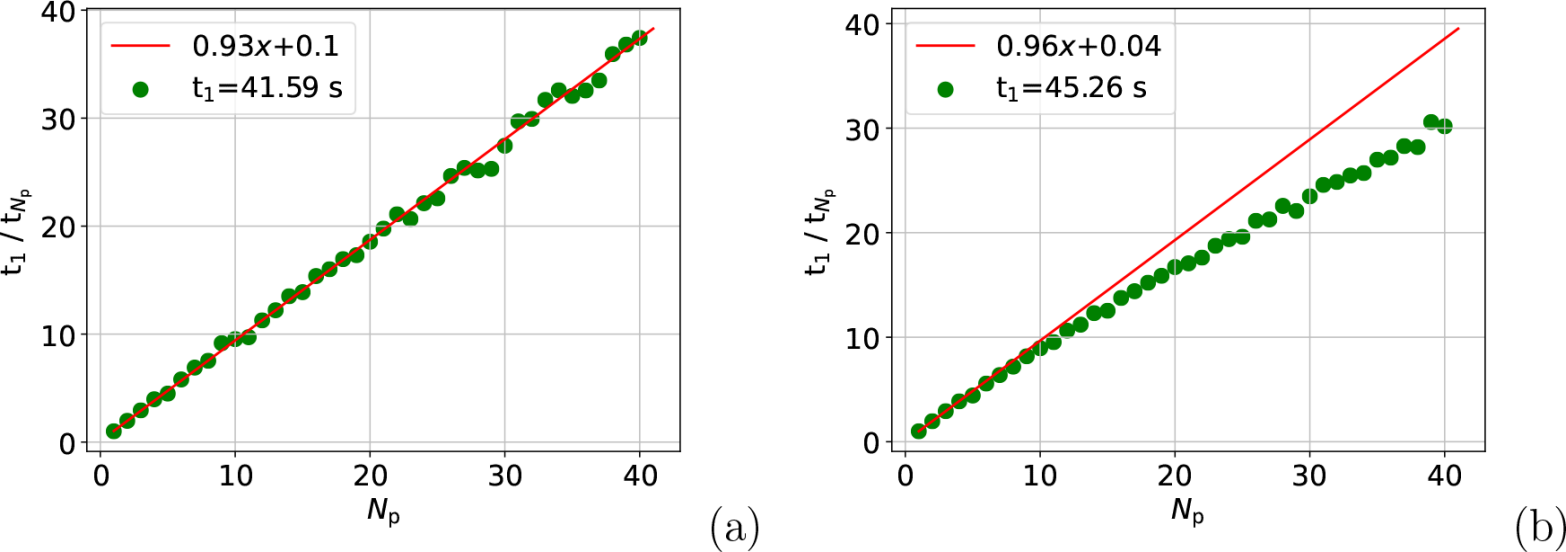
Speed-up on one node in calculating the nested triplet sum after an atom is moved (a) and for the full simulation box (b). The data in (a) are summed over 150 moves.

The time to undertake the nested sum over triplets, *t*_triplet_, in eq 9 is the main contribution to *t*_total_ and is considered in Figure 2. Figure 2(a) shows that the speed-up in *t*_triplet_ is excellent overall for the atom move calculation. Meanwhile, Figure 2(b) illustrates that the speed-up in the full box calculation is also impressive, if slightly lower. This is significant because the speed-up in *t*_triplet_ facilitates the reduction in simulation time shown in Figure 1 for both calculations. For example, for the full box calculation on a single process, *t*_triplet_ = 0.28 s, which is 85% of *t*_total_. For the atom move calculations, *t*_triplet_ is 87% of *t*_total_.

**Figure 3:**
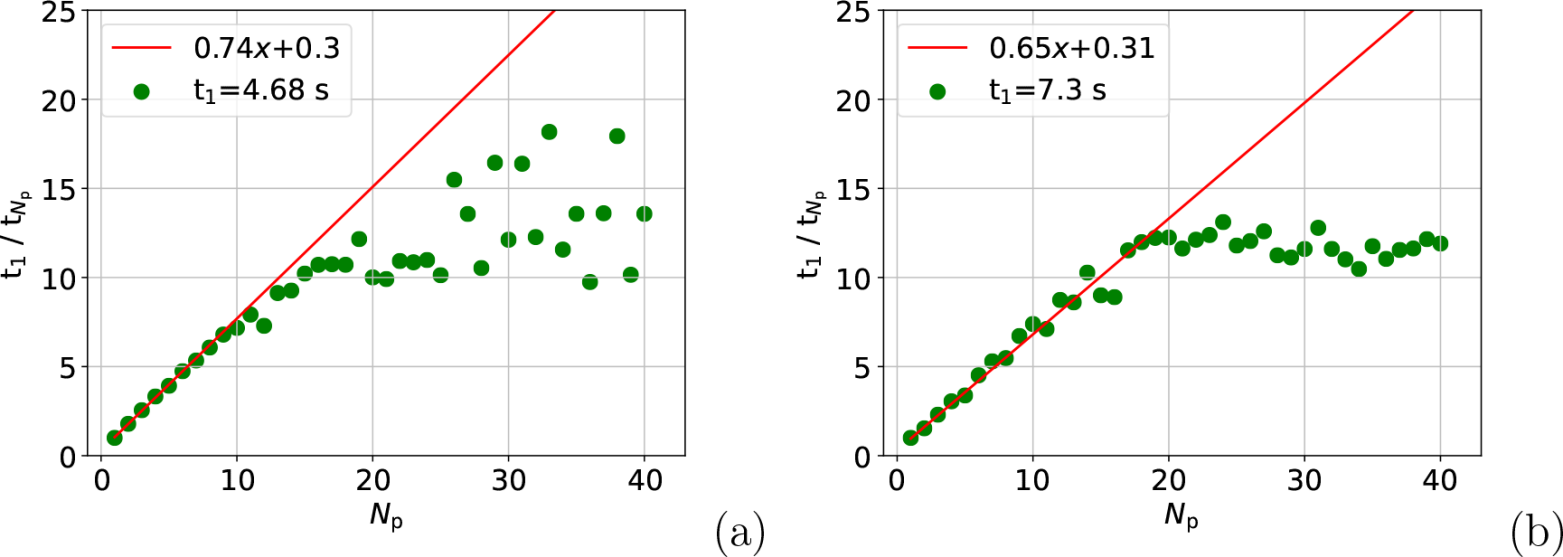
Speed-up on a single node in calculating the exponentials for the atom move (a) and the full box (b) calculations. The atom move data were summed over 150 moves.

The time required to calculate and store the exponentials in shared memory, *t*_exp_, is also a notable contribution to *t*_total_. For example, *t*_exp_ is ∼10% of *t*_total_ for the atom move calculation on a single process. For the full box calculation this rises to 14%. Figure 3 shows that an 11-fold speed-up in *t*_exp_ at *N*_p_ ≈ 15 is not bettered reliably for the atom move calculation, while for the full box calculation a plateau at a 12-fold speed-up is achieved at *N*_p_ ≈ 17. The plateaus in *t*_exp_ for both calculations partially explain the reduction in speed-up seen in Figure 1 relative to that in Figure 2. After *N*_p_ ≈ 15, *t*_exp_ is effectively a fixed cost of 0.42 and 0.61 s for the atom move and full box calculations, respectively. This means when *N*_p_ = 40, *t*_exp_ accounts for 18% of the atom move calculation and 23% of the full box calculation. Alongside the greater fixed costs associated with the full box calculation, this is the reason for the reduction in speed-up between Figure 2(b) and Figure 1(b).

**Figure 4:**
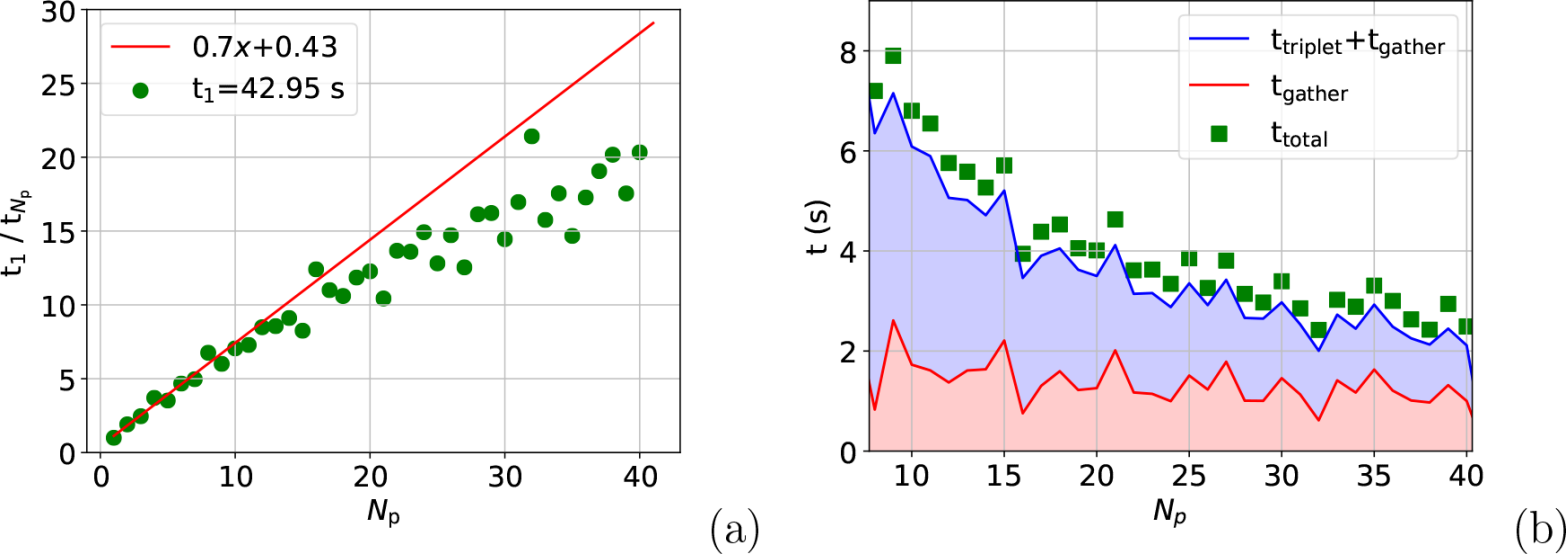
Speed-up in *t*_wait_, which is the sum of *t*_triplet_ and *t*_gather_ (a), and the values of *t*_triplet_, *t*_gather_, and *t*_total_ (b). The data are summed over 150 atom move calculations, and part (b) starts at *N*_p_ = 8.

Meanwhile, for the atom move calculation, the reduced speed-up in Figure 1 compared with Figure 2(a) is caused by the “wait” for the busiest process to send its partial sum before the total energy can be calculated. The effect of the “wait” is proven by examining the speed-up on the root process in *t*_wait_ = *t*_triplet_ + *t*_gather_, where *t*_triplet_ is the time to calculate the nonadditive energies, and *t*_gather_ is the time to add them and gather the partial sums. Figure 4 shows the speed-up in *t*_wait_ for the atom move calculation, revealing that it is similar to the speed-up seen in Figure 1. This implies that waiting for the busiest process at the gather stage is the limiting factor on the total speed-up for the atom move calculation. This assertion is reinforced by Figure 4, which shows *t*_total_ ≈ *t*_wait_.

**Figure 5:**
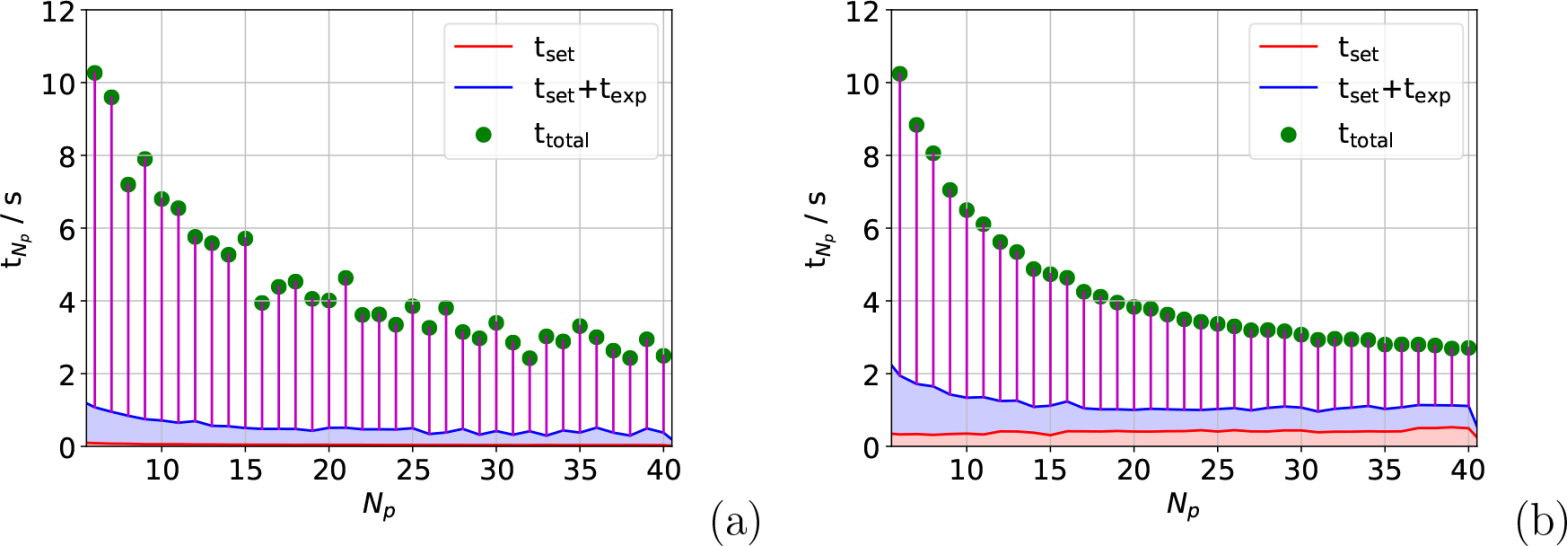
Plots showing the contributions to *t*_total_ against *N*_p_ for *N*_p_ = 6–40 for the atom move (a) and full box (b) calculations. The bottom, red section shows the contribution of the setup time *t*_set_, while the area under the blue line shows *t*_exp_. The vertical purple lines show the value of *t*_wait_ for each calculation.

Figure 5 shows the *t*_total_ breakdown into its main contributions for both calculations. The setup time *t*_set_, which is shown in red, includes all fixed costs such as instantiating arrays and process communication times. The figure shows that *t*_wait_ is the main contribution to *t*_total_ when *N*_p_ = 40 for both calculations and that the fixed costs for the atom move calculation are insignificant. Fixed costs for the full box calculation are slightly larger, however, as is the contribution of *t*_exp_.

The preceding results indicate that neither calculation had plateaued when spread over 40 processes. As such, the speed-up in both calculations across 120 processes split evenly over four nodes was investigated. The even distribution of processes means that, for example, when *N*_p_ = 120, there are 120/4 = 30 processes on each node. When *N*_p_ = 1, the process is on a single node.

**Figure 6:**
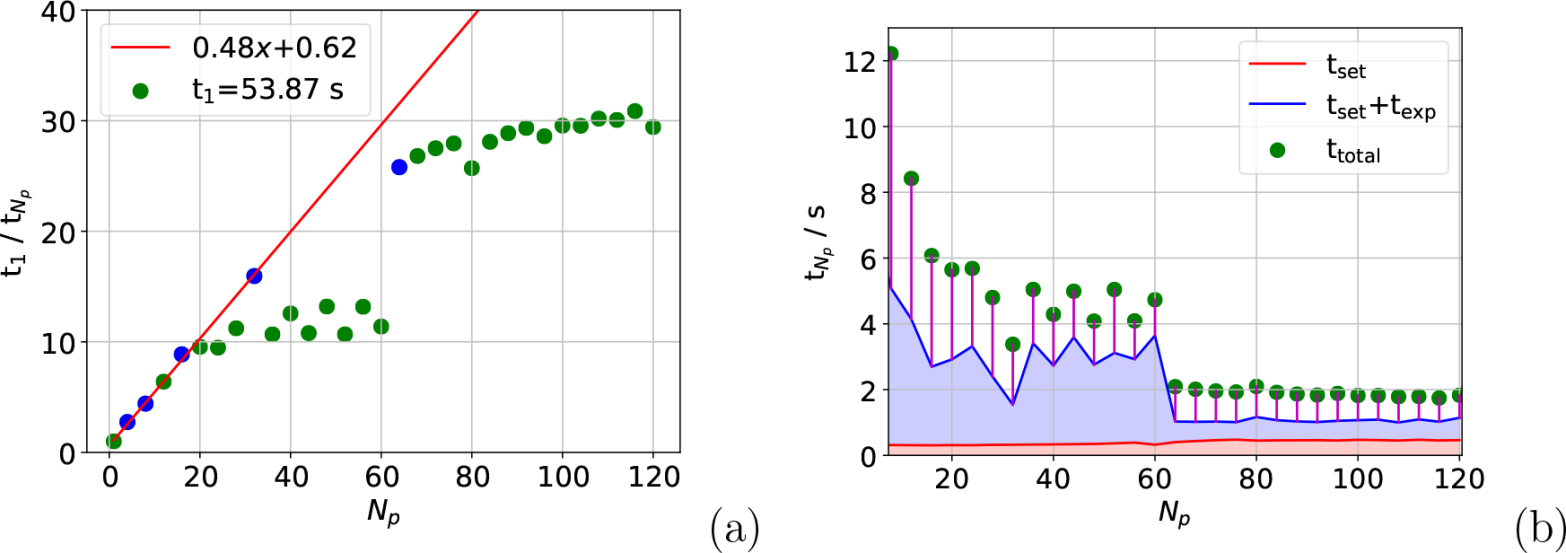
Speed-up in *t*_total_ (a) and the values of the main contributions to *t*_total_ (b) for the full box calculation on four nodes. Blue points in (a) correspond to *N*_p_ values of 2^*x*^.

The speed-up over multiple nodes for the full box calculation is displayed in Figure 6(a). A similar trend is observed over the blue points in this figure as in the overall trend in Figure 1. For example, at *N*_p_ = 32 in Figure 6(a), a roughly 17-fold speed-up is seen, which matches closely the speed-up on a single node at equivalent *N*_p_. Each blue point represents a calculation for which the total number of processes was a power of two (*i*.*e*. *N*_p_ = 4 for 2^2^, *N*_p_ = 8 for 2^3^, *etc*.). This suggests that the points on the plot where the calculation time increases with *N*_p_ are a product of the HPC architecture, rather than inherent to the algorithm. The similarity between the speed-up on four nodes and on one node for the unaffected points suggests that the algorithm extends to multiple nodes well.

The cause of the inconsistency of the parallelization of the full box calculation is evidenced in Figure 6(b), which shows that *t*_exp_ increased drastically for certain values of *N*_p_. When this happened, *t*_exp_ became the dominant cost in the calculation, degrading speed-up. The figure also shows that when *N*_p_ > 60 the setup and exponential calculation times exceed *t*_wait_. This explains the plateau in Figure 6(a).

**Figure 7:**
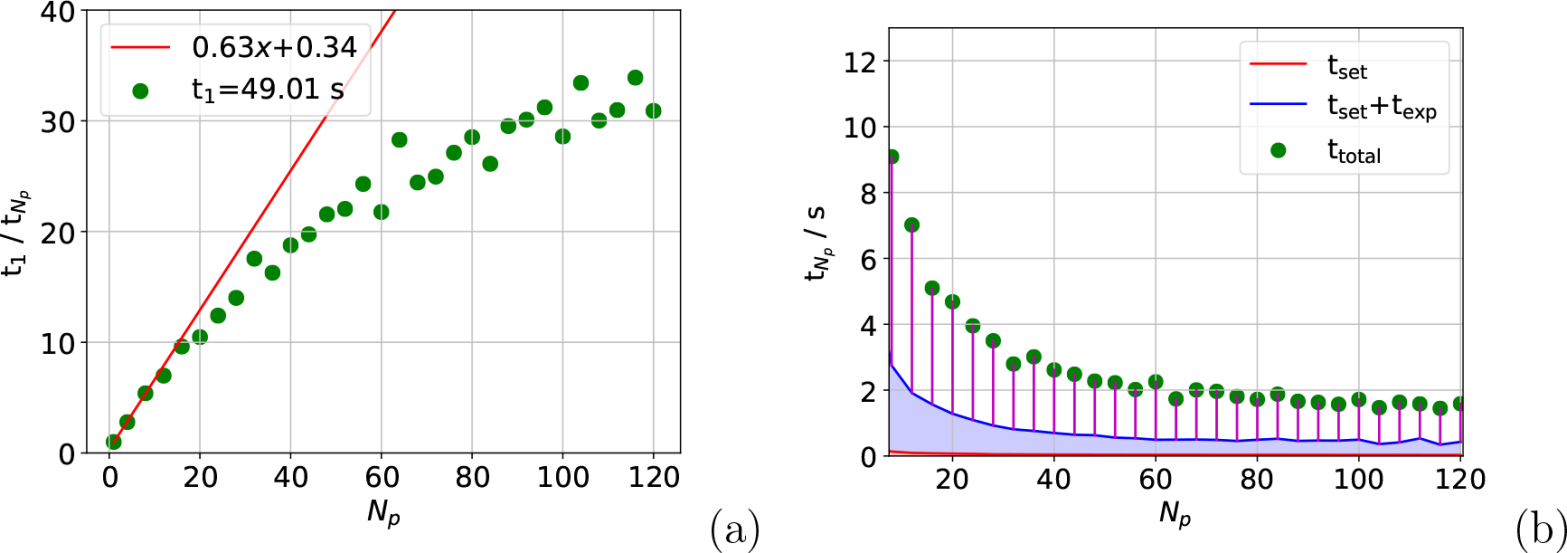
Speed-up in *t*_total_ (a) and the values of the main contributions to *t*_total_ (b) for the atom move calculations on four nodes. The calculations are spread evenly across four nodes, and the data in (a) were summed over 150 moves.

For the atom move calculation, shown in Figure 7(a), a 20-fold speed-up is achieved across 40 processes, which matches the results seen for one node in Figure 1(a). This is evidence that the calculation can be parallelized across multiple nodes without loss of performance. In addition, the distribution across more processes gives rise to a further increase in speed-up. Meanwhile, Figure 7(b) shows that *t*_wait_ nears convergence when *N*_p_ = 120. This implies that spreading the calculation over more processes is unlikely to reduce calculation time further, with the costs associated with producing and communicating between further processes having the potential to degrade speed-up.

For any calculation in which *t*_triplet_ constitutes a larger proportion of *t*_total_, such as in a system containing molecules that require a more expensive potential with more training points, a further speed-up over multiple nodes would be expected. This would be most noticeable in the full box calculation as it would eliminate the plateau shown in Figure 6(a), but it would lead to enhanced speed-up in both. The 30-fold speed-up already observed for these calculations corresponds to an eight day calculation for the simulation outlined earlier, a massive reduction from the 245 days required if no parallelization was undertaken.

## 6 Conclusions

The parallelization strategy outlined reduces significantly the calculation time for the nonadditive energy of argon. The method is robust to different computational setups, with the parallelization conferring a 20-fold speed-up on 40 processes in both calculations on one or many nodes. This corresponds to a 12 day calculation time for a first-principles-quality nonadditive energy in a simulation comprising 100,000 cycles. Distribution over more processes leads to a further reduction, with 8 days required for the calculation rather than 245 if no parallelization is attempted.

Moreover, simulations requiring a larger number of triplet calculations, such as those with large simulation boxes, would see even better parallelization. This is also true if individual triplet evaluations were more costly, as would be the case for a molecular system with a potential that requires more training points. In addition, the method for distributing the triplet nonadditive energy calculations is applicable to different potentials.

To apply the strategy to Monte Carlo simulations, long-range corrections must be applied. Additive long-range corrections are described elsewhere,^6^ while a method for implementing nonadditive corrections is given in the Supporting Information. This will allow a proof-of-concept calculation of phase coexistence in argon via a Monte Carlo simulation. Such simulations could be run for different properties or different monatomic systems using a suitable GP potential via the methods outlined above. Extension to molecular dynamics simulations is also possible by introducing forces, a method for which is also given in the Supporting Information.

Applications to systems of small molecules, such as those observed in CCUS pipelines, would require extension of the above methodology to mixtures of atomic and molecular species. This is relatively straightforward, requiring only the inclusion of an operator to identify the correct GP hyperparameters for any interaction and a method to order the interatomic distances in interactions between different species to construct the associated permutation matrices. Thus, the parallelization strategy discussed here represents a significant step toward quantitatively accurate simulations with GP potentials that give additive and nonadditive energies derived from first principles.

## Supporting Information Available

Methods to calculate forces and nonadditive long-range corrections for simulations of a single atomic species (PDF)
