# Supplementary material for: Parallel Implementation of Nonadditive Gaussian Process Potentials for Monte Carlo Simulations
Source: J Chem Theory Comput. 2023 Jun 27;19(13):4322–33. doi: 10.1021/acs.jctc.3c00113 (PMC10339671; doi:10.1021/acs.jctc.3c00113)
Supplement: Supplementary file 1 — ct3c00113_si_001.pdf [file ct3c00113_si_001.pdf]

Supporting Information: Parallel Implementation of Non-Additive Gaussian Process Potentials for Monte Carlo Simulations

Jack Broad,*,† Richard J. Wheatley,‡ and Richard S. Graham¶

†Molecular Foundry, Lawrence Berkeley National Laboratory, Berkeley, CA 94720

‡School of Chemistry, University of Nottingham, Nottingham, NG7 2RD

¶School of Mathematical Sciences, University of Nottingham, Nottingham, NG7 2RD

E-mail: jwbroad@lbl.gov

Non-additive long-range corrections

Outlined here is a method for determining the non-additive long-range correction (LRC) energy $U_{\mathrm{NA}}^{\mathrm{LRC}}$ for atomic triplets. Labelling the atoms in a triplet 1, 2 and 3 such that $r_{12} < r_{13} < r_{23}$ and denoting the cut-off as r_{c}, there are three types of triplets that require non-additive LRCs:

- $r_{12} < r_{13} < r_{\mathrm{c}} < r_{23}$, denoted Type 1.
- $r_{12} < r_{\mathrm{c}} < r_{13} < r_{23}$, denoted Type 2.
- $r_{\mathrm{c}} < r_{12} < r_{13} < r_{23}$, denoted Type 3.

Because all interacting species are atomic they can always be rotated into the same plane, though the following method could be extended to molecules regardless.

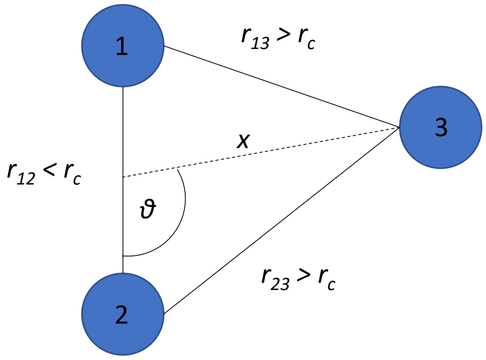

Figure 1: A sketch of a Type 2 triplet that shows the angle θ between r_{12} and the distance x.

The Type 1 scenario gives rise to a area of integration that is not trivial to define. As such, no explicit solution to this scenario is given here. Instead it is suggested that points where $r_{23}^{\mathrm{max}} = r_{13}^{\mathrm{max}} + r_{12}$ are included in the training data, to permit use of the GP potential for prediction.

Type 2

Under the Type 2 scenario, the LRC accounts for the effect of 3 on the 1-2 interaction when the former is at long-range. Thus the Type 2 LRC $U_{\mathrm{T2}}^{\mathrm{LRC}}$ can be expressed as an integral over the position of atom 3 only. In terms of interatomic distances this therefore requires integrating over both r_{13} and r_{23}. As $r_{13} < r_{23}$, atom 3 must be within the hemisphere around atom 1 that leaves it closer to 1 than 2. r_{13} must therefore be between the cut-off and infinity, whilst r_{23} must exceed r_{13} and be less than $r_{12} + r_{13}$ to obey the triangle rule.

Figure 1 shows that these distances can be re-written in terms of the angle θ and distance x. This leaves the integral as

$$U_{\mathrm{T2}}^{\mathrm{LRC}}(r_{12}) = 4\pi N_{\mathrm{a}}\rho \int_{0}^{\frac{\pi}{2}} \int_{x_{\mathrm{min}}}^{\infty} x^2 \sin\theta E^{(3)} dx d\theta. \quad (1)$$

Here, x_{min} is the value of x at which $r_{23} = r_{\mathrm{c}}$ and ρ is the density of atoms in the simulation box. The dependence of the Type 2 correction on r_{12} stems from these two atoms being

within r_c of each other. Thus this integral could be evaluated for different values of r_{12} and stored in a look-up table, which would not have to undergo an update following a volume change or exchange move.

Type 3

The Type 3 scenario is similar to Type 2 as illustrated in figure 1, with the single difference being that $r_c < r_{12}$ in the former. An equivalent to equation 1 can therefore be written down for the Type 3 correction. However, because r_{12} is now greater than the cut-off, this distance must also be integrated over. Therefore, using equivalent notation,

$$U_{\mathrm{T3}}^{\mathrm{LRC}} = \frac{4}{3}\pi^2 N_{\mathrm{a}}\rho^2 \int_{r_c}^{\infty}\int_{0}^{\frac{\pi}{2}}\int_{x_{\min}}^{\infty} r_{12}^2 x^2 \sin\theta E^{(3)} dx d\theta dr_{12}. \tag{2}$$

This integral is a purely mean field correction. Consequently, it must only be evaluated at the outset of the simulation and whenever the volume changes. The integrals in equations 1 and 2 represent a general approach to solving the long-range correction for three-body interactions as they can employ any function for $E^{(3)}$.

Calculating forces

Forces are a pre-requisite for molecular dynamics simulations. It is a useful property of GP potentials that forces can be obtained by differentiating the kernel function without the need for additional training. This process is outlined for a squared exponential kernel here.

For an atom δ, forces must be found along each of the x, y and z axes. Letting d denote an inverse interatomic distance for clarity, the force on atom δ along axis η is

$$F_\eta = -\sigma_f^2 \sum_{j\neq\delta}^{N_{\mathrm{a}}}\sum_{i=1}^{N_{\mathrm{t}}} \lambda_i \frac{\partial}{\partial \eta_\delta}\left[\exp\left(-\frac{(d_{\delta j} - d_i)^2}{2l^2}\right)\right]. \tag{3}$$

As the forces are determined from the additive energy, $N_{\mathrm{d}} = N_{\mathrm{perm}} = 1$. The sum over atoms

represents the force being an accumulation of the forces exerted by other atoms along axis η.

As d is an inverse distance, the substitution

$$v = \frac{1}{[(\mathrm{x}_\delta - \mathrm{x}_j)^2 + (\mathrm{y}_\delta - \mathrm{y}_j)^2 + (\mathrm{z}_\delta - \mathrm{z}_j)^2]^{1/2}} - d_i \tag{4}$$

can be used. Thus, writing $k = \exp\left(-\frac{v^2}{2l^2}\right)$, the derivative in equation (3) becomes

$$\frac{\partial k}{\partial \eta_\delta} = \frac{\partial k}{\partial v}\frac{\partial v}{\partial \eta_\delta}. \tag{5}$$

Letting $q = (\mathrm{x}_\delta - \mathrm{x}_j)^2 + (\mathrm{y}_\delta - \mathrm{y}_j)^2 + (\mathrm{z}_\delta - \mathrm{z}_j)^2$, one can write

$$\frac{\partial v}{\partial \eta_\delta} = \frac{\partial v}{\partial q}\frac{\partial q}{\partial \eta_\delta}. \tag{6}$$

Solving equation (6), evaluating $\frac{\partial k}{\partial v}$ and substituting the results into equation (5) gives

$$\frac{\partial k}{\partial \eta_\delta} = \frac{d_{\delta j}^3 (d_{\delta j} - d_i)(\eta_\delta - \eta_i)}{l^2} \exp\left(-\frac{(d_{\delta j} - d_i)^2}{2l^2}\right). \tag{7}$$

Thus the force along axis η is

$$F_\eta = -\sigma_f^2 \sum_{j \neq \delta}^{N_\mathrm{a}} \sum_{i=1}^{N_\mathrm{t}} \lambda_i \frac{d_{\delta j}^3 (d_{\delta j} - d_i)(\eta_\delta - \eta_i)}{l^2} \exp\left(-\frac{(d_{\delta j} - d_i)^2}{2l^2}\right). \tag{8}$$

The form of equation (8) implies that the same strategy used to calculate the energy can be applied to forces. Thus it is anticipated that the parallel speed-up will be similar for the force and energy calculations, but this has not yet been tested.

Direct training on quantum-chemical force data is also possible, with the above method suggested to reduce the number of expensive calculations necessary to train a potential for simulation. The method of differentiating the kernel function is likely to give accurate forces as the correlation length is a hyper-parameter of the model, which can be used to verify

sufficient local smoothness of the model to give confidence in the derivatives. For all of GP PES discussed in the paper the correlation lengths are consistent with derivatives being sufficiently accurate. However, quantitative testing against *ab initio* force data would be required to confirm this expectation.
